# Case of Gastrotomy for the Removal of a Leaden Bar

**Published:** 1855-07

**Authors:** 


*Case of Gastrotomy for the removal of a Leaden Bar. Recovery.* By T. B. Neal, M. D., Columbus City, Iowa.

Dear Sir—I transmit, for insertion in your valuable journal, the following remarkable and perhaps unique case.

The subject of this notice, L. Bates, at 27, resides at Wapello, twelve miles from this city. During the three days preceding Christmas last, he had been drinking freely of common whiskey; and on that day, while intoxicated, attempted, on a wager, to swallow a bar of lead. The bar was ten inches long, one-half by three-quarters of an inch thick, and weighed one pound. Thrusting it far down the œsophagus, it slipped from his grasp, and immediately entered his stomach. Dr. Bell was send for at once, but as Bates had formerly been a juggler, the doctor, thinking that he was at some of his tricks, refused to go. Bates, not much concerned at the non-attendance of the physician, worked for three days after the accident, in a pork-house, with but little inconvenience. During the night of the third day, however, he was seized with great pain in the stomach, accompanied with shooting pains along the spine, extending from the lumbar region to the sacrum, and thence to the hips. The next day he walked to Columbus, a distance of six miles, and sent for Dr. Robertson, the oldest physician in this county, to attend upon him. Dr. R. requested me to see the case with him. We found him on the fourth day, comparatively easy. His tongue was white, breath very foul, and bowels constipated. Upon careful examination, the œsophagus was found perfectly free and unobstructed. We administered to him morphia in small doses, and attempted to act upon his bowels and neutralize the poisonous effects of the lead by large doses of sulphate of magnesia. Under this treatment, although the bowels were but slightly disturbed, he was rendered astonishingly comfortable, and could walk about a little. On the third of

January, the tenth day after his accident, the severe gastric pain again returned, accompanied with vomiting, and other symptoms of gastritis.

The operation of gastrotomy was now resolved upon. Dr. Bell, of Wapello, performed the operation by making an incision through the walls of the abdomen, from the umbilicus to the false ribs, four inches in length and two inches to the left of the median line. The peritoneum being divided, Dr. Bell introduced his hand, and pushing back the protruding intestines, found that the bar of lead was nearly perpendicular, the upper end inclining a little to the left. The bar was pushed up, until the lower end came opposite the abdominal opening. It was then seized, and an incision made in the walls of the stomach, just large enough to admit of its attraction by means of forceps. The contraction of the muscular coat of the stomaeh caused the incision in the organ to close perfectly and without trouble. The external wounds was stitched, and a compress applied.

The operation was performed between three and four o'clock, P. M.; the day was cloudy, and towards sunset grew quite cold. The patient was entirely under the influence of chloroform until about two minutes before the last stitch was taken, when he revived somewhat, and expressed himself as feeling better than he had done before. When the chloroform was first administered to him, he vomited freely, hence, when the opening was made into the stomach nothing escaped therefrom, that viscus containing nothing but the leaden bar.

For the ensuing three days the system of the patient was kept under the influence of opium, and nothing but mucilaginous drinks, in small quantities, allowed as diet. He recovered as well as a patient does of uncomplicated gastritis.—*Medical Examiner*, *April*, 1855.

